# The impact of age on clinical features and fertility outcomes in patients with polycystic ovary syndrome: a secondary analysis based on the PCOSAct trial

**DOI:** 10.3389/fendo.2025.1697014

**Published:** 2026-01-13

**Authors:** Fengjuan Lu, Hong Yu, Jiaxing Feng, Yang Liu, Hang Ge, Baichao Shi, Muxin Guan, Hongli Ma, Yu Wang, Jing Cong, Wen Yang, Conghui Han, Jingshu Gao, Xiaoke Wu

**Affiliations:** 1 Graduate School, Heilongjiang University of Chinese Medicine, Harbin, China; 2 The First Affiliated Hospital of Zhejiang Chinese Medical University, Zhejiang Provincial Hospital of Chinese Medicine, Hangzhou, China; 3 The First Affiliated Hospital, Heilongjiang University of Chinese Medicine, Harbin, China; 4 Reproductive Medicine Center, Xuzhou Medical University Affiliated Lianyungang Hospital, Lianyungang, Jiangsu, China; 5 Urology Department, Xuzhou Central Hospital Southeast University, Xuzhou, Jiangsu, China

**Keywords:** age, endocrine, metabolic disorders, polycystic ovary syndrome, reproductive outcomes

## Abstract

**Objective:**

This study aimed to investigate age-related variations in baseline characteristics and reproductive outcomes among women with polycystic ovary syndrome (PCOS).

**Methods:**

A secondary analysis of the PCOSAct trial included 936 participants stratified into four age groups: 20–24, 25–29, 30–34, and 35–40 years. Differences in anthropometric measures, sex hormones, metabolic parameters, and pregnancy outcomes were analyzed using correlation and multiple logistic regression.

**Results:**

With increasing age, linear trends revealed increases in hirsutism score, systolic blood pressure, triglycerides, dyslipidemia, metabolic syndrome, and atherogenic indices (P-trend<0.05). Conversely, luteinizing hormone (LH), LH/FSH ratio, total testosterone, free testosterone, anti-Müllerian hormone (AMH), and HDL levels decreased (P-trend<0.05). Age independently correlated with these metabolic and endocrine shifts (all P<0.05). Advanced age was associated with higher ovulation induction success (adjusted OR = 1.058, 95% CI:1.006–1.114, P = 0.030) but also an increased risk of first-trimester threatened abortion (adjusted OR = 1.11, 95% CI:1.009–1.210, P = 0.031). No significant associations were observed with clinical pregnancy or live birth.

**Conclusion:**

The clinical presentation of PCOS evolves significantly with advancing age. It is typically characterized by a shift from a state of hyperandrogenism and reproductive abnormalities in younger women to a profile increasingly dominated by progressive metabolic disturbances and cardiovascular risks in later years. Although ovulation response improves in older patients, they face a higher risk of early pregnancy loss. This underscores the necessity of implementing age-stratified clinical management for individuals with PCOS.

## Introduction

Polycystic ovary syndrome (PCOS) represents one of the most prevalent endocrine disorders among women of reproductive age, with reported prevalence rates varying from 8% to 13% depending on diagnostic criteria and study populations (1). This complex condition is primarily characterized by hallmark features including polycystic ovarian morphology and clinical or biochemical hyperandrogenism, etc., which serve as key diagnostic criteria (2). The clinical presentation of PCOS frequently involves a constellation of interrelated metabolic disturbances, most notably hyperandrogenism, insulin resistance (IR) and obesity, which often coexist in affected individuals (3). Importantly, these pathological manifestations demonstrate intricate bidirectional relationships - while hyperandrogenism promotes IR, IR in turn exacerbates endocrine abnormalities. This vicious cycle contributes significantly to the progression of PCOS, driving long-term phenotypic changes, worsening endocrine-metabolic derangements, and ultimately leading to reproductive disorder including chronic anovulation and subsequent infertility (4).

PCOS exerts multisystemic pathological effects extending far beyond reproductive dysfunction, with significant metabolic consequences including dyslipidemia, hypertension, metabolic syndrome (MetS), cardiovascular diseases and type 2 diabetes (5–7). These systemic manifestations collectively contribute to substantial and enduring impacts on the lifelong health status of affected women. Notably, the clinical trajectory of PCOS exhibits distinct age-related patterns. Longitudinal studies have documented progressive ovarian functional decline in PCOS patients, characterized by diminishing ovarian volume and reduced antral follicle counts with advancing age (8). While some phenotypic features, particularly hyperandrogenic symptoms, appear to ameliorate over time (9), the other clinical manifestations remains controversial. This uncertainty is further complicated by the lack of comprehensive data elucidating potential age-specific pathological changes across different life stages. Current diagnostic frameworks, originally developed for reproductive-aged women, may be inadequate for characterizing PCOS in older populations (8). This diagnostic gap underscores the critical need for better understanding of the evolving physiological and pathological characteristics of PCOS throughout the lifespan. Such knowledge is essential for transitioning clinical management strategies from symptom-focused approaches to comprehensive, lifelong health optimization for women with PCOS.

While previous studies have noted age-related changes in PCOS, comprehensive analyses simultaneously evaluating the shift in endocrine, metabolic, and reproductive domains using large, well-characterized cohorts are limited. Furthermore, the application of novel atherogenic indices (e.g., VAI, LAP, AIP) to quantify age-dependent cardiovascular risk in PCOS remains underexplored. To address these gaps, this cross-sectional study leverages data from a large multicenter randomized controlled trial to conduct a comprehensive, age-stratified comparison. We systematically investigate four key domains: baseline clinical characteristics, sex hormone profiles, glucose-lipid metabolic parameters—including advanced atherogenic indices—and fertility outcomes. This multifaceted approach aims to provide robust, clinically relevant evidence for the age-driven evolution of PCOS phenotypes, thereby informing the development of tailored, age-specific management strategies throughout the reproductive lifespan. It is important to note that the cross-sectional design and lack of a healthy control group in this analysis limit the ability to disentangle the specific contributions of PCOS pathophysiology from the effects of physiological aging. Nevertheless, characterizing these age-associated patterns within a large PCOS cohort remains valuable for informing age-stratified clinical management.

## Materials and methods

### Target population

This study constitutes a secondary analysis of the “Polycystic Ovary Syndrome Acupuncture and Clomiphene Trial (PCOSAct).” The parent trial was a large-scale, multicenter, randomized, double-blind, controlled clinical trial conducted from 2012 to 2015 across 27 tertiary hospitals in mainland China, which enrolled 1000 infertile women with Polycystic Ovary Syndrome (PCOS) aged 20–40 years. Participants were randomized in a 1:1:1:1 ratio into four treatment groups: (1) clomiphene + active acupuncture, (2) placebo + active acupuncture, (3) clomiphene + control acupuncture, and (4) placebo + control acupuncture, with 250 participants in per group. Each group received a total of four treatment cycles. The study received ethical approval from the Institutional Review Board of the First Affiliated Hospital of Heilongjiang University of Chinese Medicine (Approval No.: 2010HZYLL-010) and was registered at ClinicalTrials.gov (Identifier: NCT01573858). Written informed consent was obtained from all participating couples prior to enrollment. Data from all participating centers were pooled and standardized according to the pre-established trial protocol to ensure consistency and reliability for this secondary analysis. Participants meeting the modified Rotterdam diagnostic criteria for PCOS were recruited (10, 11). These criteria require the presence of at least two of the following three features: oligo-ovulation or anovulation, clinical and/or biochemical hyperandrogenism, and polycystic ovarian morphology (defined ultrasonographically as the presence of ≥12 follicles measuring 2–9 mm in diameter in either ovary, or an ovarian volume ≥10 mL). Participants were excluded if their hyperandrogenism was attributed to congenital adrenal hyperplasia, Cushing’s syndrome, or androgen-secreting tumors. The detailed trial protocol and primary outcomes have been previously published (12, 13).Although 17-hydroxyprogesterone (17-OHP) was not routinely measured, potential cases of non-classical congenital adrenal hyperplasia (NCAH) were diligently screened out during the baseline evaluation by experienced reproductive endocrinologists based on clinical history, physical examination, and androgen levels, in accordance with the trial protocol. Furthermore, this study did not perform subgrouping into phenotypes A-D based on testosterone levels and ovulation status. With the core grouping principle being the age gradient, the aim was to explore the impact of age as a continuous variable on a spectrum of clinical parameters, rather than to compare static differences among PCOS phenotypes. For the present analysis, 936 participants were ultimately included and stratified into four age groups for investigation: Group 1 (20–24 years, n=146), Group 2 (25–29 years, n=507), Group 3 (30–34 years, n=256), and Group 4 (35–40 years, n=27).

### Baseline characteristics

Baseline data collection included the following parameters: (1) anthropometric parameters including height, weight, body mass index (BMI = weight [kg]/height² [m²]), waist circumference (WC), hip circumference (HC), waist-to-hip ratio (WHR = waist [cm]/hip [cm]); (2) hemodynamic measurements: systolic and diastolic blood pressure (SBP/DBP); (3) ultrasonographic evaluations including endometrial thickness (ET), volume of the both ovaries (LOV/ROV), and antral follicle count (AFC), ovarian volume calculated using the formula (length × width × thickness × π/6) (14); and (4) hirsutism score (using the Ferriman-Gallwey scoring system) (15).

### Biochemical measurements

Fasting blood samples were collected from all participants at baseline after a 12-hour overnight fast: women with regular menstrual cycles were sampled on day 3 of their cycle, while those with amenorrhea were sampled on the day of enrollment. All specimens were transported to the core laboratory at Heilongjiang University of Chinese Medicine for analysis. The following parameters were measured: serum hormonal profiles (follicle-stimulating hormone [FSH], luteinizing hormone [LH], total testosterone [TT], free testosterone [FT], anti-Müllerian hormone [AMH], sex hormone-binding globulin [SHBG]); fasting plasma glucose (FPG), fasting insulin (FINS), total cholesterol (TC), triglycerides (TG), high-density lipoprotein (HDL), low-density lipoprotein (LDL), apolipoprotein A1 (ApoA1), and apolipoprotein B (ApoB). The free androgen index (FAI) was calculated by TT (nmol/L)/SHBG (nmol/L) × 100. IR was evaluated using the homeostasis model assessment of insulin resistance (HOMA-IR = [FBG × FINS]/22.5) (16), with HOMA-IR ≥ 2.69 indicating IR (17). QUICKI = 1/(log FINS [μU/ml])+ log(FPG [mg/dl]) (18). HOMA1-IR was selected for its widespread use and comparability with prior PCOS literature, though we acknowledge that HOMA2-IR might provide additional insights. Acknowledged limitations in metabolic assessment include the lack of an oral glucose tolerance test (OGTT) and hemoglobin A1c (HbA1c) measurement, which may have resulted in under-diagnosis of dysglycemia. MetS was defined according to established criteria requiring ≥3 of the following: (1) WC >88 cm; (2) SBP ≥130 mmHg or DBP ≥85 mmHg; (3) FPG within the reference of 6.1–7.0 mmol/L (110–126 mg/dL); (4) TG ≥1.7 mmol/L (150 mg/dL); or (5) HDL <1.3 mmol/L (50 mg/dL) (12). Dyslipidemia was diagnosed based on “Chinese Guidelines for the Prevention and Treatment of Dyslipidemia in Adults”, which is formulated for the Chinese population with specific cardiovascular risk characteristics, namely: TC ≥6.22 mmol/L, TG ≥2.26 mmol/L, LDL ≥4.14 mmol/L, or HDL ≤1.04 mmol/L (19). These thresholds are consistent with Chinese population-specific guidelines and were applied to maintain clinical relevance within the study context. Lipid ratios and novel obesity indices comprised the following measures including TC/HDL, TG/HDL, APOB/APOA1, atherogenic index (AI) = (TC – HDL)/HDL (20), atherogenic index of plasma (AIP) = log10 (TG/HDL) (21), lipid accumulation product (LAP) = (WC – 58) × TG and visceral adiposity index (VAI) = [WC/(36.58 + (1.89 × BMI))] × (TG/0.81) × (1.52/HDL) (22, 23).

### Pregnancy outcomes

The study utilized the following standardized criteria for reproductive outcomes: (1) ovulation defined as serum progesterone levels >5 ng/mL; (2) biochemical pregnancy was confirmed by positive urine or serum human chorionic gonadotropin (hCG) testing; (3) clinical pregnancy was verified through transvaginal ultrasound detection of an intrauterine live fetus; (4) live birth was delivery of a viable fetus at ≥20 weeks’ gestation; (5) adverse obstetric outcomes include preeclampsia and gestational diabetes mellitus (GDM), diagnosed according to local hospital; (6) preterm birth was delivery occurring at <37 weeks’ gestation; (7) macrosomia: birth weight > 4000g.

### Statistical analyses

All statistical analyses were performed using SPSS 26.0 (IBM Corp., Armonk, NY, USA). Categorical data were expressed as frequency (%), and the chi-square test was used for comparison. Measurement data were presented as mean ± standard deviation (± s). One-way analysis of variance (ANOVA) was applied for comparisons between groups, with linear trend test conducted simultaneously, and Bonferroni method was used for multiple comparisons. Partial correlation analysis was adopted for correlation analysis. Multiple stepwise linear regression was used to analyze independent factors associated with age. Univariate binary logistic regression was performed with “yes” coded as 1 and “no” as 0 to analyze age-related fertility outcomes and perinatal outcomes, treatment modalities (acupuncture or clomiphene) from the original PCOSAct trial were included as covariates in the multivariate logistic regression models for fertility outcomes to adjust for their potential effects. The significance level was set at α = 0.05, with *P* < 0.05 indicating a statistically significant difference and *P*-trend < 0.05 suggesting a statistically significant trend difference.

## Results

### Comparison of baseline characteristics among different age groups

SBP levels and Hirsutism score increased progressively across the four age-stratified quartiles (*P*-trend <0.05), with the SBP levels of Group 4 showing markedly higher compared to Group 1 and Group 2 groups (*P* < 0.05). No other parameters exhibited statistically significant differences among groups (*P* > 0.05) (**Table 1**).

**Table 1:** Comparison of baseline characteristics among different age groups.

Variables	Group 1 (n=146)	Group 2 (n=507)	Group 3 (n=256)	Group 4 (n=27)	*P*_-trend_
Height (cm)	160.46 ± 4.96	161.41 ± 5.10	161.36 ± 5.11	160.52 ± 4.85	0.971
Weight (kg)	61.75 ± 12.34	62.66 ± 12.40	64.63 ± 12.45	63.60 ± 11.68	0.338
BMI (kg/m^2^)	23.93 ± 4.46	23.98 ± 4.15	24.78 ± 4.38	24.70 ± 3.72	0.247
Hirsutism score	2.80 ± 2.33	2.89 ± 2.76	3.30 ± 2.95	3.93 ± 3.22	0.031
WC (cm)	85.22 ± 12.16	84.85 ± 11.48	86.66 ± 11.10	87.41 ± 11.33	0.250
HC (cm)	97.37 ± 8.34	98.18 ± 8.55	99.54 ± 8.49	98.49 ± 9.55	0.383
WHR	0.87 ± 0.07	0.86 ± 0.07	0.87 ± 0.07	0.89 ± 0.09	0.230
SBP (mmHg)	110.99 ± 8.97	111.59 ± 9.46	114.21 ± 8.89	115.96 ± 9.91*^<ns/>^	0.003
DBP (mmHg)	74.55 ± 8.01	74.60 ± 7.91	75.32 ± 7.53	77.30 ± 7.19	0.070
ET (mm)	6.74 ± 1.88	6.52 ± 2.17	6.63 ± 2.21	6.44 ± 2.58	0.587
LOV (cm^3^)	10.17 ± 4.41	10.68 ± 4.71	11.60 ± 5.45	9.79 ± 3.10	0.962
ROV(cm^3^)	11.38 ± 4.75	11.64 ± 4.64	12.12 ± 7.80	10.38 ± 4.53	0.626
Left ovary’s follicle	11.95 ± 3.20	12.02 ± 2.78	12.22 ± 3.33	10.88 ± 3.51	0.129
Right ovary’s follicle	12.05 ± 3.22	12.10 ± 2.66	12.07 ± 2.79	11.56 ± 3.38	0.417

Significant differences are denoted as follows: *P<0.05 vs. Group 1; #P<0.05 vs. Group 2; &P<0.05 vs. Group 3.

### Comparison of sex hormone profiles among different age groups

The levels of LH, LH/FSH, TT, FT and AMH all showed a significant linear decline with age (*P*-trend<0.05). LH levels and LH/FSH ratios in the Group 1 group were significantly higher than those in the Group 3 and Group 4; TT levels in the Group 4 were lower than those in the Group 1, FT levels in the Group 4 were lower than those in the Group 2, and AMH levels in the Group 4 were lower than Group 1,Group 2 and Group 3(all *P* < 0.05) (**Table 2**).

**Table 2:** Comparison of sex hormone profiles among different age groups.

Variables	Group 1 (n=146)	Group 2 (n=507)	Group 3 (n=256)	Group 4 (n=27)	*P*_-trend_
FSH (mIU/mL)	6.31 ± 1.69	6.11 ± 1.65	5.92 ± 1.65	6.30 ± 2.07	0.847
LH (mIU/mL)	11.54 ± 7.12	10.77 ± 5.62	9.68 ± 5.85^*^	8.30 ± 4.76^*^	0.004
LH/FSH	1.96 ± 1.74	1.83 ± 0.99	1.65 ± 0.99^*^	1.30 ± 0.57^*^	0.003
TT (nmol/L)	1.73 ± 0.70	1.66 ± 0.61	1.66 ± 0.68	1.35 ± 0.61^*^	0.005
FT (pg/ml)	2.28 ± 0.86	2.33 ± 0.81	2.27 ± 0.86	1.84 ± 0.99^<ns/>^	0.010
FAI	6.04 ± 4.65	5.71 ± 4.29	6.08 ± 4.65	4.86 ± 2.94	0.260
AMH (ng/mL)	12.96 ± 6.29	12.30 ± 6.16	11.43 ± 6.88^*^	8.70 ± 5.30^*<ns/>&^	0.001
SHBG (nmol/L)	42.36 ± 28.55	43.37 ± 30.02	41.87 ± 32.18	38.47 ± 30.09	0.494

Significant differences are denoted as follows: *P<0.05 vs. Group 1; #P<0.05 vs. Group 2; &P<0.05 vs. Group 3.

### Comparison of glucolipid metabolic indicators among different age groups

The TG levels increased linearly with age (*P*-trend<0.001), with the Group 4 being significantly higher than the Group 1, Group 2 and Group 3(*P* < 0.05). Conversely, HDL levels decreased linearly with age (*P*-trend<0.001), and the Group 4 was significantly lower than Group 1 and Group 2 (*P* < 0.05). The incidence of dyslipidemia and MetS was highest in the Group 4, and showed a linear increase with age (*P*-trend<0.001). Indices including TC/HDL, TG/HDL, ApoB/ApoA1, AI, AIP, LAP, and VAI were all the highest in Group 4, and all showed significant linear increases with age (all *P*-trend<0.05)(**Table 3**).

**Table 3:** Comparison of glucolipid metabolism indicators among different age groups.

Variables	Group 1 (n=146)	Group 2 (n=507)	Group 3 (n=256)	Group 4 (n=27)	*P*_-trend_
FPG (mmol/L)	5.03 ± 0.98	5.05 ± 0.92	4.99 ± 1.00	5.26 ± 1.11	0.280
FINS (pmol/L)	102.50 ± 90.85	91.21 ± 81.75	95.8 ± 78.53	117.70 ± 132.28	0.347
HOMA-IR	3.51 ± 3.85	3.11 ± 3.49	3.23 ± 2.98	4.42 ± 6.14	0.199
QUICKI	0.34 ± 0.04	0.34 ± 0.05	0.33 ± 0.03	0.375	0.34 ± 0.04
IR	29 (18.7%)	92 (17.1%)	59 (21.6%)	10 (33.3%)	0.072
TC (mmol/L)	4.57 ± 1.06	4.74 ± 1.01	4.88 ± 1.22^*^	4.56 ± 1.01	0.865
TG (mmol/L)	1.47 ± 0.79	1.54 ± 0.93	1.64 ± 0.89	2.18 ± 1.16^*<ns/>&^	<0.001
HDL (mmol/L)	1.33 ± 0.35	1.29 ± 0.35	1.24 ± 0.40	1.07 ± 0.36^*<ns/>^	<0.001
LDL (mmol/L)	2.84 ± 0.88	2.96 ± 0.82	3.09 ± 0.95^*^	2.83 ± 0.78	0.835
Dyslipidemia	49(33.6%)	199 (39.3%)	125 (48.8%)^*^	20 (74.1%)^*<ns/>^	<0.001
MetS	30(19.4%)	99(18.4%)	65(23.7%)	10(33.3%)	0.045
APOA1 (g/L)	1.51 ± 0.30	1.52 ± 0.31	1.49 ± 0.32	1.39 ± 0.35	0.062
APOB (g/L)	0.84 ± 0.27	0.89 ± 0.27	0.95 ± 0.31^*^	0.92 ± 0.23	0.088
TC/HDL	3.58 ± 0.95	3.87 ± 1.15^*^	4.18 ± 1.22^*<ns/>^	4.48 ± 1.08^*<ns/>^	<0.001
TG/HDL	1.23 ± 0.84	1.36 ± 1.12	1.51 ± 1.06	2.32 ± 1.62^*<ns/>&^	<0.001
APOB/APOA1	0.57 ± 0.19	0.60 ± 0.20	0.65 ± 0.21^*<ns/>^	0.68 ± 0.16	0.004
AI	2.57 ± 0.95	2.87 ± 1.15^*^	3.17 ± 1.22^*<ns/>^	3.47 ± 1.07^*<ns/>^	<0.001
AIP	-0.00 ± 0.02	0.03 ± 0.01	0.08 ± 0.02^*<ns/>^	0.27 ± 0.05^*<ns/>&^	<0.001
LAP	39.90 ± 32.35	43.01 ± 35.33	45.00 ± 31.46	55.25 ± 44.74^*<ns/>&^	0.027
VAI	2.42 ± 1.72	2.66 ± 2.23	2.96 ± 2.12	4.65 ± 3.43^*<ns/>&^	<0.001

Significant differences are denoted as follows: *P<0.05 vs. Group 1; #P<0.05 vs. Group 2; &P<0.05 vs. Group 3.

### Partial correlation analysis between age and PCOS parameters

Partial correlation analysis (after adjusting for BMI) showed that age was positively correlated with Hirsutism score, SBP, TC, TG, ApoB, TC/HDL TG/HDL, APOB/APOA1, AI, AIP, LAP, VAI(all *P* < 0.05).While negatively correlated with AMH, LH, LH/FSH, TT, and HDL(all *P* < 0.05)(**Table 4**).

**Table 4:** Partial correlation analysis between age and PCOS parameters.

Variables	r	*P*	Variables	r	*P*
Hirsutism score	0.066	0.044	TG (mmol/L)	0.095	0.004
SBP (mmHg)	0.103	0.002	TC (mmol/L)	0.065	0.048
AMH (ng/mL)	-0.099	0.002	HDL (mmol/L)	-0.080	0.015
FSH (mIU/mL)	-0.062	0.059	LDL (mmol/L)	0.059	0.071
LH (mIU/mL)	-0.085	0.009	APOA1 (g/L)	-0.019	0.571
LH/FSH	-0.090	0.006	APOB (g/L)	0.100	0.002
TT (nmol/L)	-0.070	0.033	TC/HDL	0.114	<0.001
FT (nmol/L)	-0.047	0.156	TG/HDL	0.160	<0.001
FAI	-0.052	0.113	APOB/APOA1	0.122	<0.001
SHBG (nmol/L)	0.014	0.670	AI	0.160	<0.001
FBG (mmol/L)	0.004	0.913	AIP	0.125	<0.001
FINS (pmol/L)	-0.020	0.547	LAP	0.055	0.092
HOMA-IR	-0.011	0.743	VAI	0.122	<0.001

Adjustment factors was BMI.

### Linear regression analysis between age and clinical-biochemical characteristics in PCOS patients

Stepwise multiple linear regression assessed associations between age and the aforementioned linearly trending indicators. Age was positively correlated with SBP, hirsutism score, TC,TG, TC/HDL, TG/HDL,APOB/APOA1, AI, and VAI (all *P* < 0.05); and negatively correlated with AMH, LH, LH/FSH, and HDL(all *P* < 0.05) (**Table 5**).

**Table 5:** Correlation analysis between age and various indicators.

Variables	b	t	95% CI	*P*
SBP (mmHg)	1.812	4.299	0.985~2.639	<0.001
Hirsutism score	0.318	2.527	0.071~0.566	0.012
AMH (ng/mL)	-0.986	-3.398	-1.556~-0.417	<0.001
LH	-1.006	-3.729	-0.070~-0.004	<0.001
LH/FSH	-0.180	-3.489	-0.282~-0.079	<0.001
TC(mmol/L)	0.106	2.163	0.010~0.203	0.031
TG(mmol/L)	0.134	3.242	0.053~0.215	0.001
HDL(mmol/L)	-1.058	-3.471	-0.091~-0.025	<0.001
TC/HDL	0.301	5.795	0.199~0.403	<0.001
TG/HDL	0.212	4.275	0.115~0.309	<0.001
APOB/APOA1	0.040	4.369	0.022~0.058	<0.001
AI	0.301	5.795	0.199~0.403	<0.001
VAI	0.422	4.238	0.227~0.618	<0.001

### Comparison of pregnancy outcomes and perinatal adverse events after treatment among different age groups

Univariate binary logistic regression showed that advanced age was associated with both increased successful ovulation induction (OR = 1.053, 95% CI:1.00-1.11, *P* = 0.035) and higher first-trimester threatened abortion risk after clinical pregnancy (OR = 1.11, 95%CI:1.01-1.21, *P* = 0.025). These associations persisted after adjusting for treatment modalities (ovulation induction: adjusted OR = 1.058, 95%CI:1.01-1.11, *P* = 0.030; threatened abortion: adjusted OR = 1.11, 95%CI:1.01-1.21, *P* = 0.031). No significant associations were found for biochemical pregnancy, clinical pregnancy, live birth rate, or other pregnancy/postpartum adverse outcomes (all *P*>0.05) (**Table 6**).

**Table 6:** Comparison of pregnancy outcomes and perinatal adverse events among different age groups.

Variables	Unadjusted				After adjustment^#^			
	B	OR	95% CI	*P*	B	OR	95% CI	*P*
Ovulation	0.052	1.053	(1.004-1.106)	0.035	0.057	1.058	(1.006-1.114)	0.030
Biochemical pregnancy	-0.024	0.977	(0.937-1.018)	0.270	-0.026	0.974	(0.933-1.017)	0.237
Clinical pregnancy	-0.030	0.971	(0.926-1.018)	0.221	-0.031	0.202	(0.924-1.017)	0.969
Threatened abortion	0.102	1.108	(1.013-1.211)	0.025	0.100	1.105	(1.009-1.210)	0.031
Live Birth	-0.041	0.960	(0.915-1.008)	0.101	-0.042	0.958	(0.912-1.007)	0.090
Preeclampsia	0.128	1.136	(0.986-1.309)	0.077	0.109	1.110	(0.960-1.290)	0.144
GDM	0.072	1.074	(0.929-1.242)	0.332	0.079	1.083	(0.934-1.255)	0.293
Preterm birth	0.042	1.043	(0.876-1.240)	0.637	0.050	1.051	0.886-1.248)	0.560
Macrosomia	0.077	1.080	(0.917-1.273)	0.356	0.089	1.093	(0.925-1.290)	0.296

^#^Adjustment factors were treatment modalities.

## Discussion

Through a systematic analysis of PCOS patients stratified by different age groups, this study reveals the core driving role of age in the evolution of PCOS clinical phenotypes. The results show that the endocrine characteristics, metabolic status, and pregnancy outcomes of PCOS patients exhibit significant clinical heterogeneity with increasing age.

### Age-related changes in sex hormone profiles and ovarian reserve

This study found that levels of LH, LH/FSH ratio, TT, FT, and AMH in PCOS patients showed a significant linear downward trend with increasing age (*P*-trend<0.05), confirming the age-dependent transition of PCOS endocrine phenotypes. Among these, hyperandrogenemia, as a core pathological feature of PCOS (24), exhibits obvious heterogeneity in its age-related change patterns. In this study, TT and FT levels were significantly lower in older patients (TT in Group 4 was lower than that in Group 1, and FT in Group 4 was lower than that in Group 2, *P* < 0.05), which is consistent with the findings of Panidis et al. who observed age-related decreases in testosterone and dehydroepiandrosterone sulfate (DHEAS) (25) However, no intergroup differences were found in FAI and SHBG, which slightly differs from the trend of concurrent decreases in DHEA, DHEAS, FAI, and SHBG reported by van Keizerswaard et al, suggesting that individual differences in androgen metabolism may affect age-related change patterns (26). Hirsutism in patients with PCOS is a typical feature of hyperandrogenism. Notably, in this study, it was found that the hirsutism score did not show a linear decreasing trend with age like androgen levels; instead, it increased with age. A previous study on elderly PCOS populations (>80 years old) also demonstrated that hirsutism persisted even when androgen levels were comparable to those in healthy control groups (27). This finding contrasts with the general decline in circulating androgen levels and underscores the potential long-term sensitivity of hair follicles to androgens, a phenomenon that may persist independent of serum hormone concentrations. This dissociation between biochemical hyperandrogenism and its clinical manifestation highlights the complexity of age-related phenotypic evolution in PCOS and suggests that hirsutism is a durable marker of the syndrome’s hyperandrogenic legacy. The decrease in LH and LH/FSH ratio is another important manifestation of age-related evolution in sex hormone profiles of PCOS patients. Younger patients (Group 1) had significantly higher LH levels than Group 3, and significantly higher LH/FSH ratios than Group 3 and Group 4 (*P* < 0.05), suggesting that the state of pituitary hypersecretion in PCOS patients may gradually alleviate with increasing age. This change may be related to enhanced negative feedback regulation due to ovarian function decline, and also reflects an age-related shift in the endocrine profile, characterized by a relative decrease in LH secretion and LH/FSH ratio towards levels often seen in non-PCOS populations of similar age, providing an endocrine basis for improved responsiveness to ovulation induction in older patients.

In the assessment of ovarian reserve, both ultrasound indices (such as ovarian volume, follicle) and serum markers (such as AMH) play important roles. A previous 20-year follow-up study showed that ovarian volume in PCOS patients gradually decreases with age, remaining relatively stable before the age of 35 and decreasing significantly between 35 and 50 years (28, 29), suggesting that ovarian volume is more valuable for reflecting relatively late-stage ovarian morphological changes. Although this study observed that the ovarian volume of PCOS women in Group 4 was the smallest among the four groups, the difference did not reach statistical significance. This negative result may be related to the age characteristics of the cohort: most subjects included in this study were <35 years old with a small age span between groups, making it difficult to capture subtle changes in ovarian morphology. As the “gold standard” for assessing ovarian function, serum AMH can directly quantify the number of early follicles in the ovary (30). This study found that serum AMH levels in PCOS patients in Group 4 (the highest age group) were significantly lower than those in the other three groups (*P* < 0.05) and showed a significant negative correlation with age (r=-0.094, *P* = 0.004), with higher sensitivity than ultrasound morphological indices such as ovarian volume—despite no significant statistical difference in ovarian volume between groups in this study, AMH could accurately capture early decline in ovarian function. Based on this, for PCOS women with fertility needs, serum AMH detection can effectively assess fertility potential to guide fertility planning. However, attention should be paid to the characteristic of pathologically elevated baseline AMH levels in PCOS patients due to the pathological increase in preantral follicle count, and establishing population-specific thresholds remains a focus of future research.

### Age-related metabolic disorders: from lipid abnormalities to cardiovascular risks

The most prominent finding of this study is the negative impact of age on the metabolic status of PCOS patients, particularly reflected in the deterioration of lipid profiles and increased atherogenic risk. Results showed that with increasing age, TG in PCOS patients increased linearly (*P*-trend<0.001), while HDL decreased linearly (*P*-trend<0.001). TG levels in Group 4 were significantly higher than those in the other groups, and HDL levels were significantly lower than those in Group 1 and Group 2 (*P* < 0.05). This result is consistent with the conclusion of Falcetta et al, confirming that older PCOS patients have more significant lipid metabolism abnormalities (31). More importantly, novel metabolic risk assessment indices showed stronger age correlation: AI, AIP, and VAI all increased significantly with age (*P*-trend<0.05). Among them, the TC/HDL ratio was independently and linearly correlated with age (*P* = 0.043), and VAI increased significantly even with stable BMI, suggesting its superior utility in assessing visceral fat dysfunction and metabolic status in PCOS patients, beyond what BMI alone can reveal (32). The strong, independent association of these novel indices (VAI, AIP, LAP) with age provides a more nuanced and potentially earlier warning of cardiovascular risk stratification in the PCOS population, which may be crucial for preemptive management.

Increases in AI, AIP, and VAI can directly reflect the accumulation of atherosclerotic risk. This study observed that the incidence of dyslipidemia in the Group 4 (74.1%) was observed to be significantly higher than that in the Group 1 (33.6%) and Group 2 (39.3%) (*P* < 0.05). Additionally, the incidences of dyslipidemia and MetS both showed a linearly increasing trend with age (*P*-trend< 0.005). Combined with the results that SBP increased significantly with age (*P*-trend=0.003) and showed an independent positive correlation with age (r=0.103, *P* = 0.002), it suggests that older PCOS patients face dual metabolic burdens of hypertension and dyslipidemia—both of which are core diagnostic criteria for MetS (33). Previous studies have shown that the prevalence of MetS in PCOS patients was significantly higher than that in the general population (34), and MetS can predict the risk of cardiovascular diseases and type 2 diabetes in PCOS patients (35, 36), making age-related metabolic deterioration a key target for long-term health management in PCOS patients.

It should be noted that a major limitation of this study is the lack of a control group consisting of age-matched healthy women. Therefore, part of the age-related deterioration of metabolic parameters observed in this study (such as dyslipidemia and elevated atherogenic index) may be partially attributed to the general effects of physiological aging. However, combined with existing literature, compared with age-matched healthy women, women with PCOS experience metabolic abnormalities earlier and more severely [see relevant literature (6). In this study, relatively young PCOS patients (30–34 years old) have already exhibited significant metabolic disorders and clustering of cardiovascular risks, suggesting that the pathophysiological background of PCOS itself amplifies age-related metabolic decline. Thus, the “age-driven phenotypic evolution” described in this study should be better understood as “accelerated or exacerbated age-related changes in the context of PCOS”. This recognition emphasizes the necessity of lifelong and prospective metabolic health management for patients with PCOS.

Previous studies suggested that increasing age exacerbates IR in PCOS women, mainly driven by elevated BMI (36). In contrast, the results of our study are consistent with those of Winters SJ et al—no statistical differences in IR or BMI were found among PCOS patients in different age groups (37), and this non-significant result remained stable after adjusting for BMI. A longitudinal study further showed that in lean, normal-weight, and overweight PCOS subgroups, there was no significant association between age and IR, with only a weak correlation in the obese subgroup (BMI>30) (38). In summary, the relationship between age and IR in PCOS patients remains unclear. In addition, although androgen levels decreased with age in this study (known to improve IR), stable BMI may offset this effect, resulting in no significant change in IR. This finding provides important insights for metabolic management in PCOS: weight control may be more helpful in improving IR than simply improving androgen levels.

### Comparison with age-related changes in the general population

Interpretation Against the Backdrop of Physiological Changes in Healthy Women of Reproductive Age, placing the observed age-related changes in our PCOS cohort within the context of physiological changes in healthy women of reproductive age allows for a clearer interpretation of the impact of the disease’s pathological state on the aging process. In the general population of healthy reproductive-aged women, age-related physiological changes follow distinct patterns: ovarian reserve steadily declines with advancing age, with AMH—a key assessment marker—decreasing at an approximate rate of 0.2-0.3 ng/mL per year (39). At the endocrine level, TT levels gradually decline in middle-aged women (40), and the LH/FSH ratio also shows a mild decreasing trend (41). Regarding metabolic and cardiovascular indicators, features such as increased blood pressure and alterations in the atherogenic lipid profile (e.g., elevated TG and decreased HDL) are well-established physiological characteristics of aging in healthy women. Broadly speaking, the direction of change for these indicators with age in our PCOS cohort aligns with the general patterns observed in the healthy population, reflecting the common role of physiological aging within the PCOS patient group.

However, the key distinction lies in the fact that these changes appear to have an earlier onset and a markedly exacerbated magnitude in PCOS patients. The disease’s pathological state significantly amplifies and accelerates age-related endocrine and metabolic disturbances. In terms of ovarian reserve and endocrine profile, the decline in AMH levels from Group 1 (20–24 years) to Group 4 (35–40 years) in our PCOS cohort reached 4.26 ng/mL, far exceeding the annual decline rate seen in healthy women, indicating a significantly faster rate of ovarian reserve depletion compared to healthy peers. Metabolically, dyslipidemia was more pronounced in PCOS patients. Notably, in Group 4 (35–40 years), TG levels were 0.71 mmol/L higher and HDL levels were 0.26 mmol/L lower than in the youngest Group 1. In contrast, studies show that in healthy women, HDL begins to decline slowly before menopause (around age 50), while TG continues to rise with advancing age (42). This suggests that while certain reproductive-endocrine features of PCOS (e.g., hyperandrogenemia) may appear to attenuate with age, the disease itself accelerates the process of “metabolic aging” to a significant degree. Consequently, for individuals with PCOS, establishing a proactive and long-term metabolic monitoring system is crucial to enable early intervention and address their amplified risks for metabolic and cardiovascular diseases.

### Dual impact of age on pregnancy outcomes in PCOS patients

In terms of pregnancy outcomes, this study observed a dual role of age: older PCOS patients showed improved responsiveness to ovulation induction but increased risk of first-trimester threatened abortion. Univariate and adjusted logistic regression (adjusted for treatment modalities) showed that for each 1-year increase in age, the success rate of ovulation induction increased (OR = 1.053, *P* = 0.035; adjusted OR = 1.058, *P* = 0.030), which may be related to decreased androgen and LH levels in older patients, weakening their inhibitory effect on primordial follicles. However, the improvement in ovulation rate did not translate into better overall pregnancy outcomes—older patients had a significantly increased risk of first-trimester threatened abortion (OR = 1.11, *P* = 0.025; adjusted OR = 1.11, *P* = 0.031), which is partially consistent with previous reports that “older PCOS patients have poorer pregnancy and live birth rates” (43). The potential mechanism underlying this paradox may be related to age-related decline in oocyte quality and metabolic abnormalities. This study confirmed that older PCOS patients simultaneously have a higher incidence of lipid abnormalities and an increased risk of threatened abortion. Multiple studies have demonstrated that lipid abnormalities can affect the normal development of oocytes and embryo quality through multiple mechanisms such as oxidative stress, inflammatory activation, vascular endothelial dysfunction, and hormonal metabolic disorders, leading to an increased proportion of aneuploid embryos and a decreased proportion of high-quality embryos, while also impairing endometrial receptivity (44, 45) A study by Zeyneloglu HB indicated that preconception lipid-lowering interventions in women with dyslipidemia can significantly improve clinical pregnancy rates and ongoing pregnancy rates (46), suggesting that optimizing lipid metabolism should be a key component of assisted reproductive therapy for older PCOS patients. Notably, this study found no significant association between age and clinical pregnancy rate, live birth rate, or other perinatal adverse events (such as preeclampsia, gestational diabetes mellitus), which may be related to the small sample size (especially only 27 cases in Group 4) and confounding effects of interventions. Further verification with large-sample studies are needed in the future. Although this study adjusted for covariates related to treatment modalities (acupuncture plus placebo or sham acupuncture plus clomiphene) in the analysis of fertility outcomes, it did not thoroughly explore the interaction effects between treatment measures and age. The impact of different interventions on ovulation and pregnancy outcomes may vary across PCOS patients of different ages. Therefore, the associations within this dimension require further detailed analysis in subsequent research.

### Strengths and limitations

The strengths of this study include its multicenter design, large overall sample size, and comprehensive assessment of age-related characteristics in PCOS patients, covering multiple dimensions such as endocrine, metabolic, and pregnancy outcomes. This provides solid data support for age-stratified management of PCOS. However, several limitations of this study should be acknowledged. First, the cross-sectional design precludes the determination of causal relationships between age and the evolution of PCOS phenotypes. Second, the absence of an age-matched control group of women without PCOS. It prevents us from quantifying how much of the observed age-related variation is specific to the pathophysiology of PCOS versus attributable to the universal process of physiological aging in women. Third, the sample size was highly unbalanced across age groups, with the oldest cohort (Group 4, 35–40 years) comprising only 27 participants. This limits the statistical power for this subgroup, increases the risk of Type II error, and precludes the generalization of findings related to older reproductive-age women with PCOS. The results pertaining to Group 4 should therefore be interpreted as preliminary and require validation in larger, dedicated cohorts. Fourth, several potential confounding factors, such as smoking status, dietary habits, physical activity levels, and specific medication use, were not systematically collected or adjusted for in the analysis. Based on these limitations, we propose specific directions for future research. Prospective longitudinal cohort studies with extended follow-up periods are essential to delineate the causal pathways of phenotypic evolution in PCOS. Future studies should include healthy control groups to clarify the disease-specific nature of these age-related changes. Efforts should be made to recruit larger cohorts of older PCOS patients to validate our findings. Furthermore, integrating data on genetics, lifestyle, and environmental exposures will be crucial for developing a more holistic understanding of the factors driving the progression of PCOS across the lifespan.

### Conclusion

In summary, this study demonstrates that age is significantly associated with the evolution of clinical phenotypes in PCOS, significantly modulating endocrine profiles, metabolic risk, and reproductive performance. With advancing age, there is a clear transition from a reproductive-hyperandrogenic phenotype in younger patients toward a predominantly adverse metabolic and cardiovascular risk profile in later reproductive years. Notably, although older women with PCOS exhibited a more favorable ovulatory response, they faced a heightened risk of early pregnancy loss, underscoring a clinically important reproductive–metabolic disconnect. These findings highlight the critical importance of adopting age-stratified management strategies in PCOS, emphasizing earlier metabolic surveillance in younger women and vigilant obstetric support in older patients. Future longitudinal studies with matched control groups are warranted to confirm these trends and clarify the underlying mechanisms, ultimately guiding personalized, lifespan-oriented care for women with PCOS.

## Data Availability

The raw data supporting the conclusions of this article will be made available by the authors, without undue reservation.
